# ABT199/venetoclax potentiates the cytotoxicity of alkylating agents and fludarabine in acute myeloid leukemia cells

**DOI:** 10.18632/oncotarget.28193

**Published:** 2022-02-10

**Authors:** Benigno C. Valdez, David Murray, Bin Yuan, Yago Nieto, Uday Popat, Borje S. Andersson

**Affiliations:** ^1^Department of Stem Cell Transplantation and Cellular Therapy, University of Texas MD Anderson Cancer Center, Houston, TX 77030, USA; ^2^Division/Department of Experimental Oncology, University of Alberta/Cross Cancer Institute, Edmonton T6G 1Z2, Alberta, Canada

**Keywords:** ABT199/venetoclax, busulfan, cyclophosphamide, fludarabine, acute myeloid leukemia

## Abstract

The antineoplastic activity of pre-transplant regimens in hematopoietic stem cell transplantation (HSCT) is a critical factor for acute myeloid leukemia (AML) patients. There is an urgent need to identify novel approaches without jeopardizing patient safety. We hypothesized that combination of drugs with different mechanisms of action would provide better cytotoxicity. We, therefore, determined the synergistic cytotoxicity of various combinations of the alkylating agents busulfan (Bu) and 4-hydroperoxycyclophosphamide (4HC), the nucleoside analog fludarabine (Flu) and the BCL2 inhibitor ABT199/venetoclax in AML cells. [Bu+4HC] and [Bu+Flu] inhibited cell proliferation and activated apoptosis; addition of ABT199 to either combinations significantly increased these effects with combination indexes < 1. Apoptosis is suggested by cleavages of PARP1 and CASPASE 3, DNA fragmentation, increased reactive oxygen species, decreased mitochondrial membrane potential, and increased pro-apoptotic proteins in the cytoplasm. A similar enhancement of apoptosis was observed in patient-derived cell samples. ABT199/venetocalx upregulated anti-apoptotic MCL1 as a compensatory mechanism but addition of [Bu+4HC] or [Bu+Flu] negated this effect by CASPASE 3-mediated cleavage of MEK1/2 and its substrate MCL1. CASPASE 3 caused cleavage of pro-survival β-CATENIN, which likely contributed to the activation of stress signaling pathways involving SAPK/JNK and AMPK. The observed synergistic cytotoxicity was associated with an inhibition of pro-survival pathways involving STAT1, STAT5 and PI3K. These findings will be useful in designing clinical trials using these drug combinations as pre-transplant conditioning regimens for AML patients.

## INTRODUCTION

Allogeneic hematopoietic stem cell transplantation (allo-HSCT) provides a high rate of curability for acute myeloid leukemia (AML) patients. Its success depends in part on the efficacy of the pre-transplant conditioning regimen, which usually includes chemotherapeutic agents such as alkylating drugs. The combination of busulfan (Bu) and cyclophosphamide, two DNA alkylators, is an effective regimen [1], but is associated with a high treatment-related mortality rate [2].

To maintain efficacy and improve safety, Bu has instead been combined with nucleoside analog(s) including fludarabine (Flu). The antineoplastic activity of Bu and Flu involve different mechanisms; Bu forms DNA adducts/crosslinks while Flu gets incorporated into newly-replicating DNA strands during DNA synthesis, and they transiently arrest the cell cycle at G2- and S-phase, respectively [3, 4]. Both processes trigger a chain of events including induction of DNA strand breaks and subsequent activation of pro-apoptotic pathways [4–6]. A combination of Bu and Flu is an effective pre-transplant regimen for HSCT for AML patients, provided that the drugs are sequenced properly [4, 7–9].

There is an urgent need to improve the efficacy of the pre-transplant regimen without jeopardizing patient safety. This can be accomplished by judiciously including novel agent(s) with different mechanisms of action. One such candidate drug is ABT199/venetoclax, a BH3-mimetic small molecule that binds to and inhibits the anti-apoptotic B-cell lymphoma 2 (BCL2) protein, preferentially causing malignant cells to undergo apoptosis [10]. Preclinical studies have demonstrated the cytotoxicity of ABT199/venetoclax in leukemia cells [11]. We recently reported that ABT199 potentiated the cytotoxicity of a [gemcitabine+Bu+melphalan] combination in multiple myeloma cells [12]. ABT199 is FDA-approved when combined with azacitidine or decitabine for the treatment of AML patients ≥ 75 years of age [13, 14]. The effects of ABT199 in combination with alkylating agents and nucleoside analogs in AML cells have not been reported.

Here, we investigated the *in vitro* effects of ABT199 when combined with [Bu+4-hydroperoxycyclophosphamide] or [Bu+Flu] in AML cells. Synergistic cytotoxicities were observed for both of the three-drug combinations in AML cell lines and in patient-derived cell samples. Our results may be of importance for the design of clinical trials using [alkylating agent(s)±nucleoside analog(s)+ABT199] combinations as part of the pre-transplant conditioning regimen for AML patients undergoing allo-HSCT.

## RESULTS

### Combination of ABT199 with [Bu+4HC] or [Bu+Flu] exerts synergistic cytotoxicity towards AML cell lines

**Figure 1:**
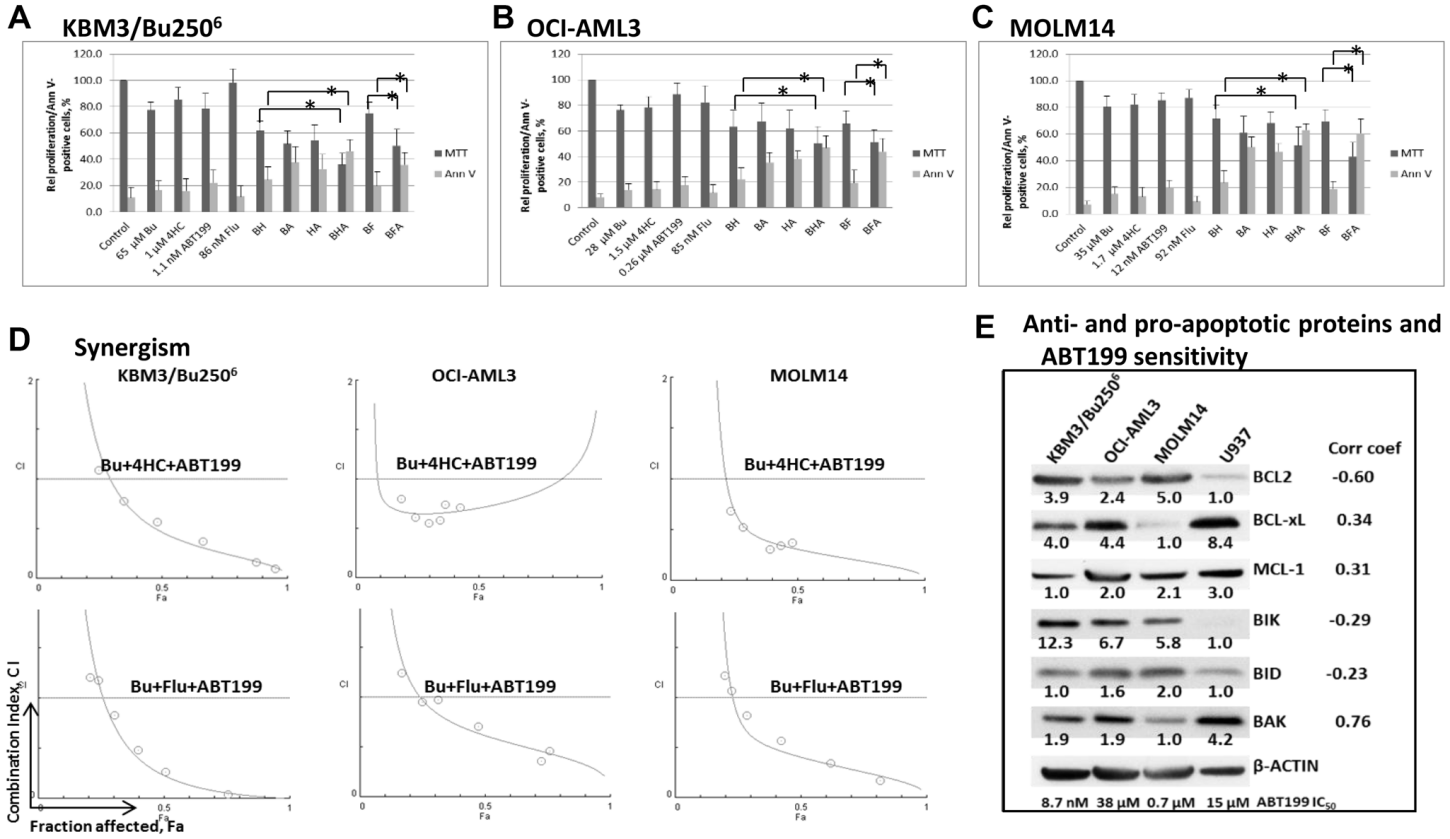
Cytotoxicity of ABT199 in combination with alkylating agents and the nucleoside analog fludarabine. (**A**–**C**) AML cells were exposed to the indicated concentrations of busulfan (Bu or B), 4- hydroperoxycyclophosphamide (4HC or H), ABT199 (A) and fludarabine (Flu or F), alone or in combination, for 48 h prior to determination of relative cell proliferation by the MTT assay and apoptosis by the Annexin V (Ann V) assay. Asterisks (^*^) indicate a statistically significant difference (*P* < 0.05) between drug combinations with and without ABT199. The results are averages of at least three independent experiments. (**D**) To determine drug synergism, cells were exposed to various drug combinations at constant ratio concentrations for 48 h prior to MTT assay. The relationships between the calculated combination indices (CI) and fraction affected (Fa) are shown below the bar graphs. CI <1 indicates synergism. The graphs are representatives of two independent experiments. (**E**) Western blotting was used to determine the correlation between the levels of anti- and pro-apoptotic proteins and the concentration of ABT199 that caused 50% inhibition of cell proliferation (IC_50_). Correlation coefficients (Corr coef) are indicated in the right-hand column.

Combinations of Bu with either cyclophosphamide or fludarabine are the most commonly-used pre-transplant conditioning regimens for AML patients [8]. They are very similar in their antileukemic activity, although the double-alkylator regimen clinically appears more toxic [19, 20]. Using drug concentrations close to their IC_20_ values, we determined if addition of ABT199/venetoclax would potentiate their cytotoxicity towards AML cells. Continuous exposure of KBM3/Bu250^6^ cells for 48 h to 65 μM Bu, 1 μM 4HC, 1.1 nM ABT199 or 86 nM Flu resulted in ~22%, ~14%, ~21%, and ~2% inhibition of cell proliferation, respectively, relative to control cells (Figure 1A). The two-drug combinations [Bu+4HC], [Bu+ABT199], [4HC+ABT199] and [Bu+Flu] increased the inhibitory effects to ~38%, ~48%, ~46%, and ~25%, respectively, and exposure to the three-drug combinations [Bu+4HC+ABT199] and [Bu+Flu+ABT199] resulted in ~64% and ~50% inhibition of cell proliferation (Figure 1A).

The observed drug-mediated effects on cell proliferation are consistent with the increased annexin V-positivity, an indicator of apoptosis activation. The background level of annexin V-positivity in untreated control cells was ~9%. Exposure of KBM3/Bu250^6^ cells to Bu, 4HC, ABT199 or Flu resulted in ~17%, ~16%, ~22%, and ~12% annexin V-positive cells, respectively (Figure 1A). [Bu+4HC], [Bu+ABT199], [4HC+ABT199] and [Bu+Flu] increased annexin V-positivity to ~24%, ~38%, ~32%, and ~20%, respectively, while [Bu+4HC+ABT199] and [Bu+Flu+ABT199] exposure further increased the effects to ~46% and ~36% (Figure 1A).

Similar effects were observed in two other AML cells lines. Exposure of OCI-AML3 to 28 μM Bu, 1.5 μM 4HC, 0.26 μM ABT199 or 85 nM Flu resulted in ~24%, ~22%, ~12%, and ~18% inhibition of cell proliferation, respectively, and ~14%, ~14%, ~18%, and ~12% annexin V-positivity compared to ~6% in untreated control cultures (Figure 1B). Exposure to the two-drug combinations [Bu+4HC], [Bu+ABT199], [4HC+ABT199] and [Bu+Flu] inhibited cell proliferation by ~37%, ~32%, ~38%, and ~34%, respectively, and increased annexin V-positivity to ~22%, ~35%, ~38%, and ~19%. The inhibition of proliferation further increased to ~50% and ~49% following exposure to the three-drug combinations [Bu+4HC+ABT199] and [Bu+Flu+ABT199], with the proportion of annexin V-positive cells increasing to ~47% and ~44% (Figure 1B). Exposure of MOLM14 cells to 35 μM Bu, 1.7 μM 4HC, 12 nM ABT199 or 92 nM Flu resulted in ~20%, ~18%, ~15%, and ~13% inhibition of cell proliferation, respectively, and ~15%, ~13%, ~20%, and ~9% annexin V-positivity compared to ~5% in untreated control cultures (Figure 1C). Exposure to the two-drug combinations [Bu+4HC], [Bu+ABT199], [4HC+ABT199] and [Bu+Flu] inhibited cell proliferation by ~29%, ~39%, ~32%, and ~30%, respectively, and increased annexin V-positivity to ~24%, ~50%, ~47%, and ~19%. The three-drug combinations [Bu+4HC+ABT199] and [Bu+Flu+ABT199] inhibited proliferation of MOLM14 cells by ~49% and ~57% respectively, and increased annexin V-positivity to ~63% and ~61% (Figure 1C).

Addition of ABT199 to either [Bu+4HC] or [Bu+Flu] therefore significantly increased the inhibition of cell proliferation and annexin V-positivity in cultures of all three cell lines as indicated by the calculated *P* values and indicated by asterisks in Figure 1A–1C. To assess whether these effects represent synergistic interactions, cells were exposed to different concentrations of individual drugs or to the three-drug combination at a constant concentration ratio followed by analysis with the MTT assay after 48 h and the data were analyzed using the Chou-Talalay method [18]. Combination index (CI) values at increasing drug effects were graphically analyzed as shown in Figure 1D. At 50% inhibition of cell proliferation, or 0.5 fraction affected (Fa), the calculated CI values for [Bu+4HC+ABT199] were 0.4, 0.7 and 0.3 in the KBM3/Bu250^6^, OCI-AML3 and MOLM14 cell lines, respectively. CI values of 0.2, 0.6 and 0.4 were calculated for KBM3/Bu250^6^, OCI-AML3 and MOLM14 cells, respectively, following exposure to [Bu+Flu+ABT199] (Figure 1D). Strong synergism (CI <1) of the three drugs was therefore indicated in each cell line.

### Negative correlation between the level of BCL2 protein and sensitivity to ABT199

The wide range of ABT199 concentrations (1.1 nM – 260 nM) used in the cytotoxicity assays reflects the broadly differing sensitivities of the three AML cell lines used in the study to this drug. Previous studies by Pan and colleagues [11] indicated that the sensitivity of a panel of AML cell lines to ABT199 (based on IC_50_ values) correlated with their levels of BCL2 protein, whereas BCL-xL protein levels were inversely correlated with ABT199 sensitivity. We therefore determined whether there was a similar correlation between the IC_50_ values of ABT199 and the cellular levels of its target BCL2 protein or other anti- and pro-apoptotic proteins in four AML cell lines. Figure 1E shows the correlation coefficients of −0.6, 0.34, 0.31, −0.29, −0.21, and 0.76 for BCL2, BCL-xL, MCL1, BIK, BID, and BAD, respectively. These findings indicate a strong negative correlation between ABT199 IC_50_ values and the level of its target BCL2 protein. These results are consistent with previously published observations on AML, T-ALL and neuroblastoma cell lines [11, 21, 22] but not with our data obtained in cultured myeloma cell lines [12].

### [Bu+4HC+ABT199] and [Bu+Flu+ABT199] combinations activate multiple biomarkers of apoptosis

**Figure 2:**
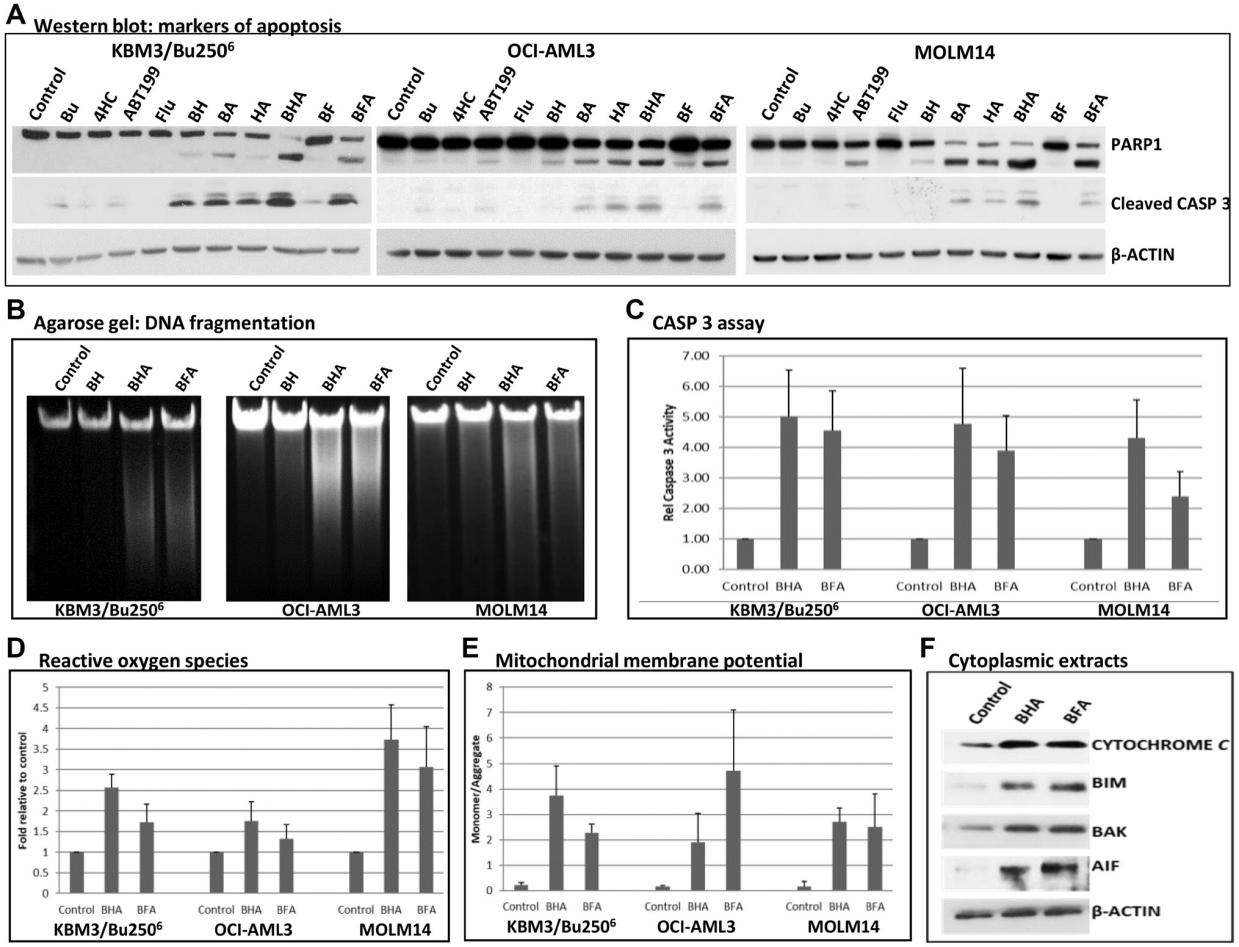
Effect of individual and combined drugs on the status of molecular markers of apoptosis. Cells were exposed to the indicated drugs for 48 h, harvested, and analyzed by (**A**) Western blotting, (**B**) DNA agarose gel electrophoresis, (**C**) CASPASE 3 enzymatic assay, and by flow cytometric analysis to determine (**D**) ROS production and (**E**) status of MMP. (**F**) KBM3/Bu250^6^ cells were exposed to solvent or the indicated drugs for 48 h and used to isolate cytoplasmic extracts, which were analyzed by Western blotting. CASP 3 = CASPASE 3; other abbreviations are the same as in Figure 1.

The increase in annexin V-positivity seen in cells exposed to the three-drug combinations indicates activation of apoptosis (Figure 1A–1C). These results are consistent with the observed cleavage of PARP1 and CASPASE 3 in KBM3/Bu250^6^, OCI-AML3 and MOLM14 cells treated with both of the three-drug combinations (Figure 2A). Activation by cleavage of CASPASE 3 is known to activate nuclear DNases that mediate DNA fragmentation, a biochemical hallmark of apoptosis [23]. In fact, agarose gel analysis of genomic DNA isolated from cells exposed to the three-drug combinations confirmed an increase in DNA fragmentation (Figure 2B). To further analyze the importance of caspases in drug-mediated cell death, we determined the enzymatic activity of CASPASE 3 in cells exposed to the three-drug combinations. Exposure of the three cell lines to [Bu+4HC+ABT199] and [Bu+Flu+ABT199] resulted in an ~2.5–5-fold increase in CASPASE 3 activity relative to the control cells (Figure 2C).

To better understand the cellular responses underlying drug-mediated cell death, we examined the production of reactive oxygen species (ROS), which are known cell-death mediators. Exposure of KBM3/Bu250^6^ cells to [Bu+4HC+ABT199] increased ROS production by ~2.5-fold relative to the control, and exposure to [Bu+Flu+ABT199] increased ROS levels by ~1.7-fold (Figure 2D). Less dramatic results were observed in OCI-AML3 cells, where exposure to [Bu+4HC+ABT199] and [Bu+Flu+ABT199] increased ROS levels by ~1.7-fold and 1.3-fold, respectively. More significant differences were observed in MOLM14 cells; exposure to individual or two-drug combinations resulted in a ~1–2-fold increase in ROS levels while exposure to [Bu+4HC+ABT199] and [Bu+Flu+ABT199] increased ROS levels by ~3.7-fold and ~3-fold, respectively. These results suggest that the drug combinations have perturbed the mitochondrial metabolism, resulting in enhanced generation of ROS.

To substantiate the effects of the three-drug combinations on the integrity of the mitochondria, their impact on the mitochondrial membrane potential (MMP) was determined using JC-1 reagent as a surrogate marker/tracer. A higher ratio of monomeric JC-1 to its aggregated form would indicate leakage of JC-1 from the mitochondria into the cytoplasm, suggesting a decreased MMP. As shown in Figure 2E, exposure of the three cell lines to [Bu+4HC+ABT199] resulted in monomer/aggregate ratios of ~1.9–3.8; exposure to [Bu+Flu+ABT199] similarly resulted in ratios of ~2.2–4.8 (Figure 2E). These marked increases in comparison to the control cells are indicative of a major decrease in the cellular MMP.

The observed depolarization of the mitochondrial membrane would indicate leakage of mitochondrial proteins, including pro-apoptotic factors, into the cytoplasm. Exposure of KBM3/Bu250^6^ cells to both of the three-drug combinations increased the levels of CYTOCHROME c, BIM, BAK and AIF proteins in the cytoplasmic extracts (Figure 2F). Overall, these results suggest extensive depolarization of the mitochondrial membrane in cells exposed to the three-drug combinations, causing the leakage of pro-apoptotic mitochondrial factors into the cytoplasm, thereby initiating the caspase-dependent cascade of events leading to apoptosis.

### Effects of [Bu+4HC+ABT199] and [Bu+Flu+ABT199] combinations on patient-derived cells

**Figure 3:**
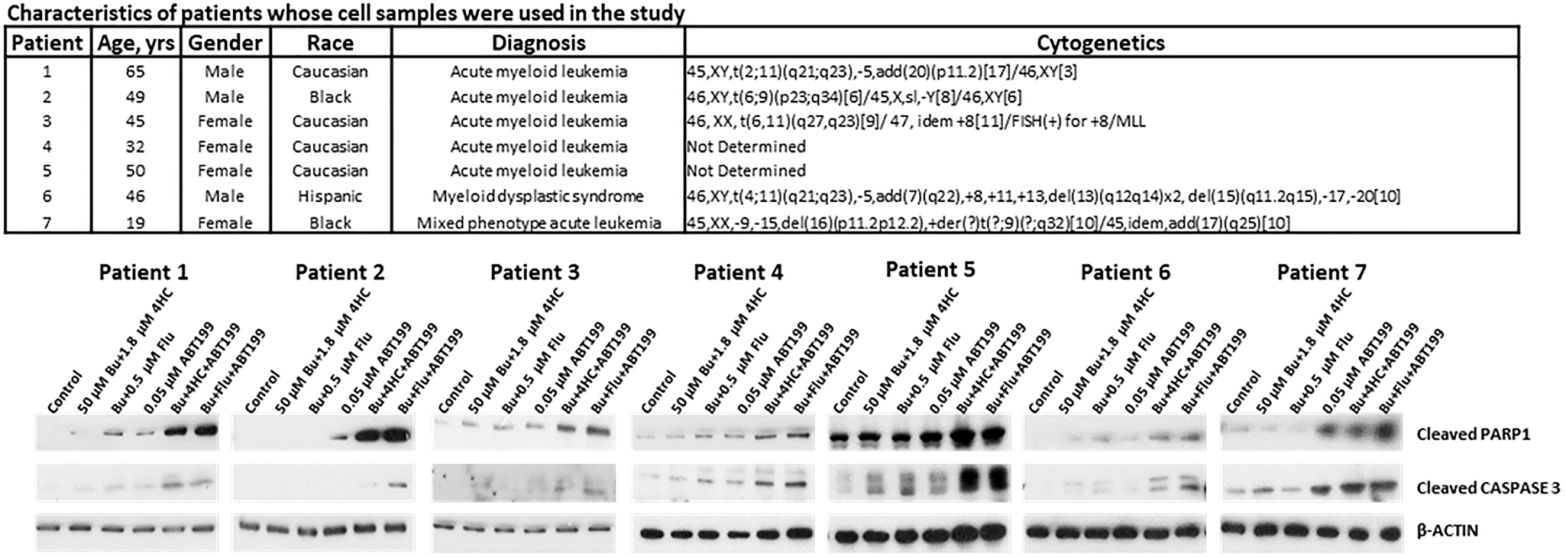
Drug-mediated induction of apoptosis in patient-derived cell samples. Mononuclear cells were isolated from peripheral blood of seven patients with hematological malignancies and exposed to the indicated drugs for 48 h prior to Western blot analysis. The upper panel shows key characteristics of the patients.

To assess the potential clinical implications of our results, we isolated mononuclear cells (MNCs) from peripheral blood of patients with acute leukemia and myeloid dysplastic syndrome and exposed the cells to drugs. Figure 3 (upper panel) shows the characteristics of the patients whose MNCs were used in this study. Exposure of the isolated MNCs to [Bu+4HC], [Bu+Flu] or ABT199/venetoclax (lanes 2, 3, and 4, respectively) resulted in minimal cleavages of PARP1 and CASPASE 3 relative to the control cells (lane 1) but exposure to [Bu+4HC+ABT199] or [Bu+Flu+ABT199] (lanes 5 and 6) substantially increased the level of their cleavage product (Figure 3), consistent with what was seen in the three established AML cell lines (Figure 2A). These observations suggest that the three-drug combinations are highly cytotoxic to patient-derived leukemic cells.

### [Bu+4HC+ABT199] and [Bu+Flu+ABT199] combinations increase CASPASE 3-mediated cleavage of MCL1 and MEK1/2

**Figure 4:**
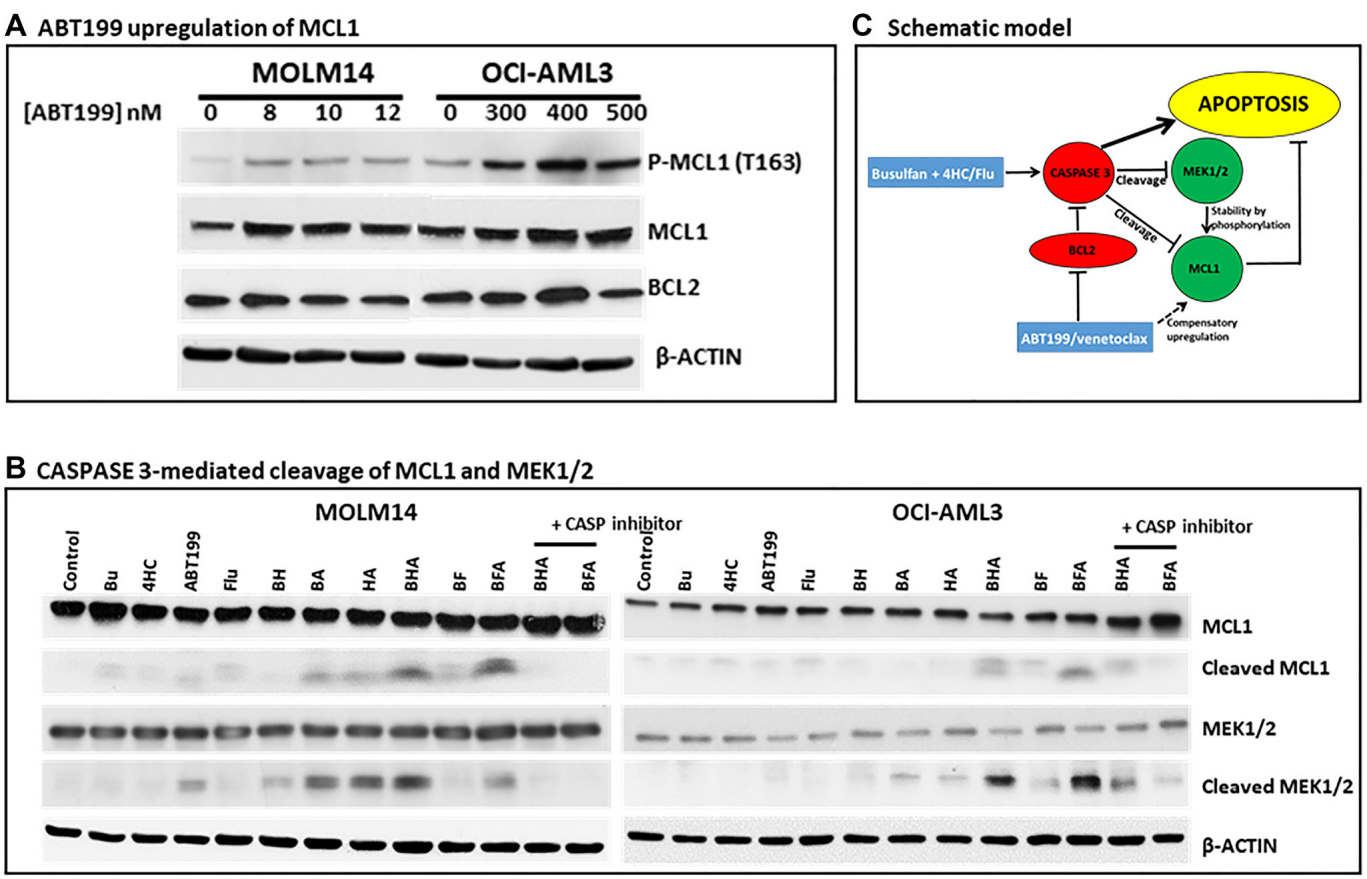
Effect of drugs, alone or in combination, on MCL1 and MEK1/2 cleavage. (**A**) Cells were exposed to increasing concentrations of ABT199 (A) or to the indicated drug(s) (**B**) for 48 h and analyzed by Western blotting. (**C**) Schematic diagram showing a model that outlines a proposed mechanism for the observed synergistic cytotoxicity of ABT199 with DNA alkylators and fludarabine. Z-VAD-FMK (40 μM) was used to inhibit caspases.

ABT199/venetoclax inhibition of BCL-2 has been reported to result in compensatory upregulation of the anti-apoptotic MCL1 in lymphoma [24] and neuroblastoma cell lines [22]. We, therefore, determined if this effect occurs in our cell line models. Exposure of MOLM14 and OCI-AML3 cells to increasing concentrations of ABT199 increased the level of phosphorylated and total MCL1 protein and decreased the level of BCL-2 protein at higher concentrations (Figure 4A). Since phosphorylation of MCL1 by MEK1/2 is known to stabilize the protein [25], and since CASPASE 3 catalyzes the inactivation by cleavage of MCL1 [26] and MEK1/2 [27], we hypothesized that the observed triple-drug-mediated activation of CASPASE 3 (Figure 2C) might have contributed to their synergistic cytotoxicity. To this effect, exposure of MOLM14 and OCI-AML3 cells to [Bu+4HC+ABT199] and [Bu+Flu+ABT199] increased the cleavage of MCL1 and MEK1/2, and addition of the CASPASE 3 inhibitor Z-VAD-FMK significantly decreased this cleavage (Figure 4B, last two lanes). Overall, these results indicate that the observed drug synergism is partly due to a concerted increase in CASPASE 3 activity which negates the compensatory upregulation of MCL1 (Figure 4C).

### [Bu+4HC+ABT199] and [Bu+Flu+ABT199] combinations activate stress signaling pathways

**Figure 5:**
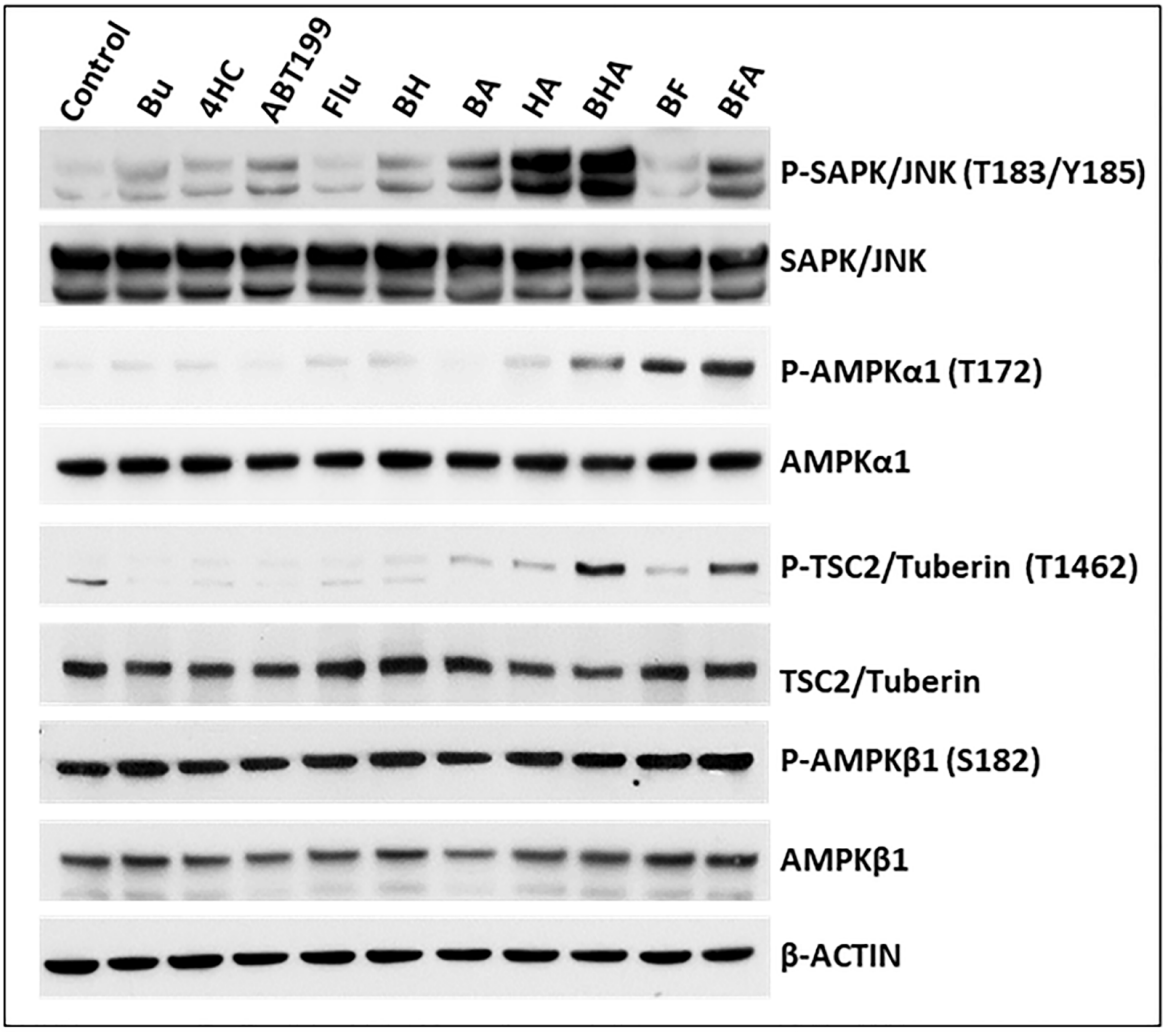
Effect of drugs, alone or in combination, on the status of various proteins involved in stress-activated signaling pathways. OCI-AML3 cells were exposed to solvent (control) or the indicated drug(s) for 48 h prior to Western blot analysis. Abbreviations are the same as in Figure 1.

The observed depolarization of mitochondria in cells exposed to [Bu+4HC+ABT199] and [Bu+Flu+ABT199] may cause drug-mediated activation of stress signaling pathways that promote apoptosis. The SAPK/JNK signal transduction pathway is known to convert stress signaling into apoptotic signaling [28]. Western blot analysis of three-drug-treated OCI-AML3 cells showed increased activation by phosphorylation of SAPK/JNK at threonine 183/tyrosine 185 (Figure 5). Another key mediator of stress signaling pathways is AMP-activated protein kinase (AMPK), a protein that monitors shifts in the cellular AMP/ADP to ATP ratio and becomes activated by decreased MMP and/or increased ROS [29, 30]. Figure 5 shows a significant increase in the phosphorylation of AMPKα1 at threonine 172 in OCI-AML3 cells exposed to both of the three-drug combinations; phosphorylation of AMPKβ1, however, was not affected, suggesting isoform specificity. An increase in the phosphorylation of TSC2/Tuberin, a known substrate for AMPKα1 [31], was also observed following the three-drug exposures. All of these findings indicate that activation of stress signaling pathways may contribute to the cytotoxicity of [Bu+4HC+ABT199] and [Bu+Flu+ABT199].

### [Bu+4HC+ABT199] and [Bu+Flu+ABT199] combinations down-regulate pro-survival pathways

**Figure 6:**
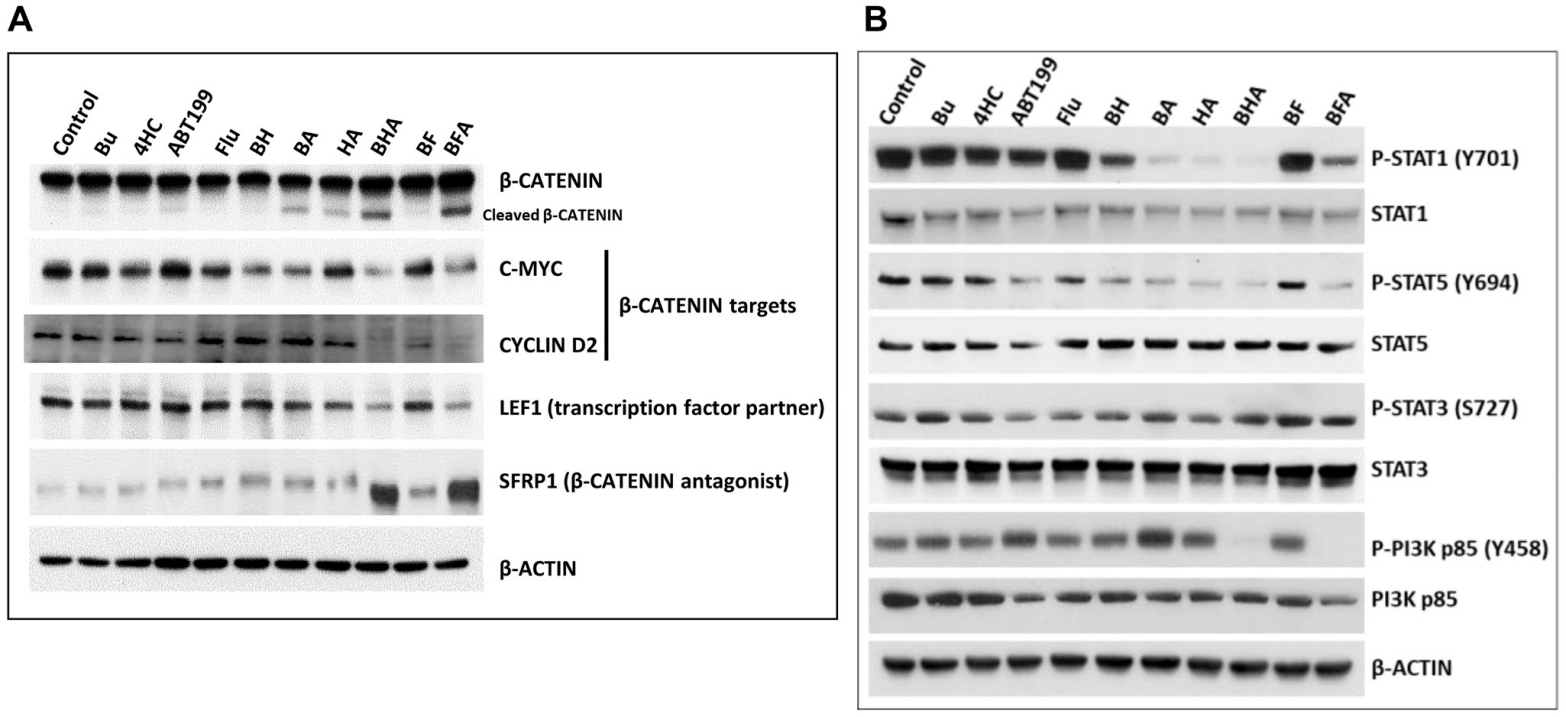
Effect of drugs, alone or in combination, on the status of various proteins involved in cellular pro-survival signaling pathways. OCI-AML3 cells were exposed to solvent (control) or the indicated drug(s) for 48 h prior to Western blot analysis using antibodies against proteins involved in the Wnt/β-catenin (**A**) and STAT and PI3K (**B**) signaling pathways.

The observed activation of CASPASE 3 (Figure 2) might also have targeted other proteins involved in pro-survival pathways, such as β-CATENIN [32]. We, therefore, determined the effects of the three-drug combinations on the WNT/β-CATENIN pathway which is constitutively activated in some hematological malignancies [33]. Western blot analysis showed cleavage of β-CATENIN in OCI-AML3 cells exposed to both of the triple-drug combinations (Figure 6A). These effects correlate with a decrease in the expression of the β-CATENIN-target genes including c-MYC [34] and CYCLIN D2 [35], as shown by a decrease in their protein levels in cells exposed to the three-drug combinations (Figure 6A). The protein level of LEF1, a known transcription factor partner of β*-*CATENIN [36], also decreased in cells exposed to both of the triple-drug combinations while the level of SFRP1 protein, a β*-*CATENIN antagonist [37] increased (Figure 6A). These results indicate that the cytotoxicity of the three-drug combinations is partly due to inhibition of the pro-survival WNT/β-CATENIN signal transduction pathway.

We also sought to determine the effects of the three-drug combinations on the status of activation by phosphorylation of the STAT and PI3K proteins, which are essential in the maintenance and survival of malignant myeloid cells [38, 39]. Exposure of OCI-AML3 cells to [Bu+4HC] decreased the phosphorylation of STAT1 at tyrosine 701; this effect was much greater after exposure to [Bu+ABT199] or [4HC+ABT199] and was even more pronounced in cells exposed to [Bu+4HC+ABT199] (Figure 6B). The [Bu+Flu] combination did not affect the level of P-STAT1 (Y701), but this parameter was significantly decreased when ABT199 was added to this combination (Figure 6B). Similar drug-mediated effects were observed on the phosphorylation of STAT5 at tyrosine 694 but not on the phosphorylation of STAT3 at serine 727.

Since there is known crosstalk between the STAT5 and PI3K pathways [40], we also determined the effects of the three drugs alone or in combination on the level of phosphorylated PI3K. Exposure of OCI-AML3 cells to any individual drug or two-drug combination did not cause a significant change in the phosphorylation status of the regulatory subunit (p85) of PI3K at tyrosine 458; however, both the [Bu+4HC+ABT199] and [Bu+Flu+ABT199] combinations significantly decreased the level of P-PI3K p85 (Y458) (Figure 6B). These results suggest that the cytotoxicity of [Bu+4HC+ABT199] and [Bu+Flu+ABT199] combinations may be partially attributed to the inhibition of pro-survival pathways involving STAT1, STAT5 and PI3K.

## DISCUSSION

This study demonstrates a marked potentiation of the cytotoxicity of [Bu+4HC] and [Bu+Flu] when combined with the BCL2 inhibitor ABT199/venetoclax in the KBM3/Bu250^6^, OCI-AML3 and MOLM14 established AML cell lines. Similar effects were observed in leukemic blast cells isolated from peripheral blood of leukemia patients, suggesting a generally enhanced anticancer efficacy of the [Bu+4HC+ABT199] and [Bu+Flu+ABT199] combinations. The cytotoxicity of the two-drug combinations [Bu+4HC] and [Bu+Flu] is primarily mediated by drug-induced DNA damage resulting in CASPASE 3 activation, as we previously reported [17, 41]. The synergistic potentiation of the effects of these two-drug combinations by ABT199/venetoclax is partly due to CASPASE 3-mediated cleavages of MEK1/2 and MCL1.

ABT199/venetoclax selectively binds to and inhibits BCL2 but not other anti-apoptotic proteins including MCL1 and BCL-xL. However, a compensatory upregulation of MCL1 is observed in cells exposed to ABT199/venetoclax, probably due to its stabilization by phosphorylation catalyzed by MEK1/2 [22, 24]. Since both MCL1 and MEK1/2 are substrates for CASPASE 3, this compensatory mechanism is negated by a concerted increase in the activity of CASPASE 3 in cells exposed to the triple-drug combinations.

Increased CASPASE 3 activity might also have mediated the cleavage of the pro-survival β-CATENIN protein. When activated in the cytoplasm, β-CATENIN translocates to the nucleus and mediates transcription of multiple pro-survival genes [36, 42]. The observed increase in CASPASE 3 activity in cells exposed to the three-drug combinations correlates with cleavage of β*-*CATENIN and down-regulation of its downstream targets (Figure 6A). Overall, these results suggest that the synergism of the [Bu+4HC+ABT199] and [Bu+Flu+ABT199] combinations may be partially attributed to the inhibition of the WNT/β*-*CATENIN pathway.

The extensive perturbation of the mitochondrial membrane in cells exposed to [Bu+4HC+ABT199] and [Bu+Flu+ABT199] was not only associated with upregulation of CASPASE 3 but also with activation of stress-signaling pathways that promote apoptosis. For example, the SAPK/JNK signal transduction pathway is a known conduit to apoptotic signaling [28]. The increased phosphorylation of SAPK/JNK at threonine 183/tyrosine 185 in cells exposed to the three-drug combinations (Figure 5) is consistent with the activation of this pathway. AMPKα1 is another stress sensor that is important in maintaining intracellular homoeostasis [43]. The genotoxic stress mediated by [Bu+4HC+ABT199] and [Bu+Flu+ABT199] likely activated AMPKα1 by increased phosphorylation at threonine 172, consistent with increased phosphorylation of its known substrate TSC2/Tuberin at threonine 1462. Interestingly, phosphorylation of AMPKβ1 at serine 182 was not affected (Figure 5). Induction of these stress signaling pathways presumably played a role in the increased activation of apoptosis seen with these three-drug combinations.

These findings are consistent with the observed inhibition of other pro-survival pathways, which are normally activated by phosphorylation of key proteins. STAT1 and STAT5 are constitutively activated in AML cells [44], consistent with the observed high levels of P-STAT1 (Y701) and P-STAT5 (Y694) in control OCI-AML3 cells (Figure 6B). Exposure to [Bu+4HC] or [Bu+Flu] decreased their levels, and the effects were further enhanced when ABT199 was added to either combination (Figure 6B), indicating that the pro-survival JAK/STAT pathway involving STAT1 and STAT5 must have been compromised. Moreover, the observed inhibition of phosphorylation of the p85 regulatory subunit of PI3K at tyrosine 458 (Figure 6B) is consistent with the known crosstalk between the JAK/STAT and PI3K pathways at the transcriptional and posttranslational levels in leukemia cells [40, 45].

In summary, our results indicate strong antineoplastic activity of [Bu+4HC+ABT199] and [Bu+Flu+ABT199] towards AML cells. The genotoxic stress imposed by the alkylating agents and Flu and the inhibition of the anti-apoptotic BCL2 protein by ABT199 was associated with an activation of stress signaling pathways and inhibition of pro-survival signaling pathways, leading to an enhanced activation of the intrinsic apoptotic pathway. The results from this preclinical study may be used as the basis for clinical trials using [Bu+4HC+ABT199] or [Bu+Flu+ABT199] as pre-transplant conditioning therapy for high-risk AML patients undergoing allo-HSCT.

## MATERIALS AND METHODS

### Cell lines and drugs

KBM3/Bu250^6^ is a Bu-resistant AML cell line established in our laboratory [15]. The OCI-AML3 and MOLM14 AML cell lines were kindly provided by Dr. Michael Andreeff’s laboratory (University of Texas MD Anderson Cancer Center, Houston, TX, USA), and the U937 cell line was obtained from the American Type Culture Collection (Manassas, VA, USA). Cells were grown in RPMI 1640 medium (Mediatech, Manassas, VA, USA) supplemented with 10% heat-inactivated fetal bovine serum (FBS: Atlanta Biologicals, Flowery Branch, GA, USA) and 100 IU/mL penicillin and 100 μg/mL streptomycin (Mediatech) at 37°C in a humidified atmosphere of 5% CO_2_ in air. Busulfan was obtained from Sigma-Aldrich (St. Louis, MO, USA) and dissolved in dimethyl sulfoxide (DMSO) immediately prior to each experiment. Fludarabine and ABT199 were purchased from Selleck Chemicals LLC (Houston, TX, USA) as 10 mM solutions in DMSO and stored at −20°C. 4-Hydroperoxycylophosphamide (4HC) was a generous gift from Dr. Scott Rowley (Hackensack University Medical Center, Hackensack, NJ, USA). For *in vitro* studies, 4HC is commonly used in place of cyclophosphamide, which is a stable pro-drug requiring enzymatic activation. In an *in vitro* setting 4HC is spontaneously converted to 4-hydroxycyclophosphamide, which is further converted to the active metabolites, phosphoramide mustard and acrolein [16].

### Drug cytotoxicity assays

The cytotoxicity of drugs alone or in combination was determined as previously described using the 3-(4,5-dimethylthiazol-2-yl)-2,5-diphenyl tetrazolium bromide (MTT) assay [17]. Cell proliferation was determined relative to control cells exposed to solvent alone. Apoptosis was determined by flow-cytometric measurements of phosphatidylserine externalization with Annexin-V-FLUOS (Roche Diagnostics, Indianapolis, IN, USA) and 7-aminoactinomycin D (BD Biosciences, San Jose, CA, USA) using a Muse Cell Analyzer (EMD Millipore, Burlington, MA, USA). Drug combination effects were estimated based on the combination index (CI) values [18] calculated using the CalcuSyn software (Biosoft, Ferguson, MO, USA). This program was developed based on the median-effect method: CI <1 indicates synergy, CI ~1 indicates additivity, and CI >1 suggests antagonism.

### Western blot analysis

Cells exposed to drugs or solvent for 48 h were collected by centrifugation, washed with ice-cold PBS and lysed with cell lysis buffer (Cell Signaling Technology, Danvers, MA, USA). The total protein concentrations were determined using a BCA Protein Assay kit (ThermoFisher Scientific, Rockford, IL, USA). Proteins were resolved on polyacrylamide-SDS gels and blotted onto nitrocellulose membranes (Bio-Rad, Hercules, CA, USA). Western blot analyses were done by chemiluminescence using the Immobilon Western Chemiluminescent HRP Substrate (EMD Millipore). The sources of the antibodies and their optimum dilutions are provided in Supplementary Table 1.

### Analysis of DNA fragmentation and determination of CASPASE 3 activity

Cells were exposed to drugs for 48 h, harvested and washed with ice-cold PBS. A fraction of the washed cells was used to isolate genomic DNA using the Wizard^®^ Genomic DNA Purification Kit (Promega, Madison, WI, USA) and DNA fragmentation was determined using 0.8% agarose gel electrophoresis in Tris-HCl borate+EDTA buffer containing ethidium bromide. The remaining washed cells were used to prepare total cell extracts using the CASPASE 3 Colorimetric Activity Assay kit (Chemicon International, Temecula, CA, USA). Total protein concentration was determined as described above. Equal amounts of protein were analyzed for CASPASE 3 activity using the same kit. Fold changes in CASPASE 3 activity were calculated relative to the control cell extracts.

### Analysis of reactive oxygen species

Cells exposed to solvent or drugs for 48 h were analyzed for production of ROS using 5-(and-6)-chloromethyl-2′,7′-dichlorodihydrofluorescein diacetate, acetyl ester (CM-H_2_DCFDA), an ROS indicator that diffuses into cells where it is oxidized to a fluorescent product (Life Technologies, Grand Island, NY, USA). Briefly, cells exposed to drugs were aliquoted (0.5 mL) into 5 mL tubes and 3 μL of 1.5 mM CM-H_2_DCFDA (dissolved in DMSO) was added. Cells were incubated at 37°C for 1 h and immediately analyzed with a Gallios Flow Cytometer (Beckman Coulter, Brea, CA, USA) using excitation/emission wavelengths of 492/520 nm. Geometric means of the fluorescence intensities were compared and the fold increase in ROS production relative to control was calculated.

### Analysis of mitochondrial membrane potential

An MMP detection kit (Cayman Chemical Co., Ann Arbor, MI, USA) was used to determine changes in MMP using the 5,5′,6,6′-tetrachloro-1,1′,3,3′-tetraethylbenzimidazolylcarbocyanine iodide (JC-1) reagent. Cells were exposed to solvent or drugs for 48 h and aliqouted (0.5 mL) into 5 mL tubes. Diluted (1:10 with cell growth medium, 4 μL) JC-1 fluorescent dye was added to each tube, incubated at 37°C for 20 min, and immediately analyzed by flow cytometry as described by the manufacturer.

### Preparation of cytoplasmic extracts

Cells were exposed to drugs as described above. Cytoplasmic extracts, prepared using the Mitochondria Isolation Kit for Cultured Cells - Reagent-based method (ThermoFisher Scientific), were mixed with loading buffer and dithiothreitol (Cell Signaling Technology) and boiled for 5 min. Denatured protein samples were analyzed by Western blotting.

### Statistical analysis

Results are presented as the mean ± standard deviation of at least three independent experiments and statistical analysis was performed using Student’s paired *t*-test with a two-tailed distribution (Microsoft Excel, Redmond, WA, USA). The same software was used to determine the correlation coefficients.

## SUPPLEMENTARY MATERIALS



## Abbreviations

AML: acute myeloid leukemia
Ann V: annexin V
Bu: busulfan
CI: combination index
CM-H_2_DCFDA: 5-(and-6)-chloromethyl-2′,7′-dichlorodihydrofluorescein diacetate: acetyl ester
DMSO: dimethyl sulfoxide
Flu: fludarabine
h: hours
4HC: 4-hydroperoxycyclophosphamide
HSCT: hematopoietic stem cell transplantation
JC-1: 5,5′,6,6′-tetrachloro-1,1′,3,3′-tetraethylbenzimidazolylcarbocyanine iodide
min: minutes
MMP: mitochondrial membrane potential
MNCs: mononuclear cells
MTT: 3-(4,5-dimethylthiazol-2-yl)-2,5-diphenyl tetrazolium bromide
ROS: reactive oxygen species
