# Supplementary material for: ABT199/venetoclax potentiates the cytotoxicity of alkylating agents and fludarabine in acute myeloid leukemia cells
Source: Oncotarget. 2022 Feb 10;13:319–30. doi: 10.18632/oncotarget.28193 (PMC8830224; doi:10.18632/oncotarget.28193)
Supplement: Supplementary file 1 [file oncotarget-13-28193-s001.pdf]

ABT199/venetoclax potentiates the cytotoxicity of alkylating agents and fludarabine in acute myeloid leukemia cells

SUPPLEMENTARY MATERIALS

Table 1: List of primary antibodies, their sources and dilutions

Antigen	Company/Cat.#	Source	Dilution*
β-ACTIN	Sigma/A5316	Mouse	6000
AIF	Cell Signaling/5318	Rabbit	2000
P-AMPKα1 (T172)	Cell Signaling/50081	Rabbit	1500
AMPKα1	Cell Signaling/5831	Rabbit	1500
P-AMPKβ1 (S182)	Cell Signaling/4186	Rabbit	2000
AMPKβ1	Cell Signaling/4150	Rabbit	3000
BAK	Cell Signaling/6947	Rabbit	2000
BCL2	Cell Signaling/4223	Rabbit	2000
BCL-xL	Cell Signaling/2764	Rabbit	3000
BID	Cell Signaling/2002	Rabbit	2500
BIK	Cell Signaling/4592	Rabbit	2000
BIM	Cell Signaling/2933	Rabbit	2500
β-CATENIN	Cell Signaling/8480	Rabbit	2500
Cleaved CASPASE 3	Cell Signaling/9661	Rabbit	2500
Cleaved PARP1 (Asp214)	Cell Signlaing/5625	Rabbit	2000
CYCLIN D2	Cell Signlaing/3741	Rabbit	1800
CYTOCHROME c	BD PharMingen/556433	Mouse	1500
LEF1	Cell Signaling/2230	Rabbit	2000
MCL1	Santa Cruz/819	Rabbit	1000
P-MCL1 (T163)	Cell Signaling/14765	Rabbit	2500
MEK1/2	Cell Signaling/8727	Rabbit	2500
c-MYC	Cell Signaling/5605	Rabbit	3000
PARP1	Santa Cruz Biotech/8007	Mouse	1000
P-PI3K p85 (Y458)	Cell Signaling/4228	Rabbit	2500
PI3K p85	Cell Signaling/4257	Rabbit	2000
P-SAPK/JNK (T183/Y185)	Cell Signaling/4668	Rabbit	2000
SAPK/JNK	Cell Signaling/9258	Rabbit	2500
SFRP1	Cell Signaling/3534	Rabbit	1800
P-STAT1 (Y701)	Cell Signaling/7649	Rabbit	2000
STAT1	Cell Signaling/9172	Rabbit	3000
P-STAT3 (S727)	Cell Signaling/9134	Rabbit	2000
STAT3	Cell Signaling/9132	Rabbit	2000
P-STAT5 (Y694)	Cell Signaling/9359	Rabbit	2000
STAT5	Santa Cruz Biotech/835	Rabbit	700
P-TSC2/TUBERIN (T1462)	Cell Signaling/3617	Rabbit	2500
TSC2/TUBERIN	Cell Signaling/4308	Rabbit	4000

*Used anti-rabbit IgG for secondary antibody from Bio-Rad Lab. Used anti-mouse IgG for secondary antibody from Bio-Rad Lab. **Fold dilution in PBS with 0.05% Tween 20.
